# Role of lncRNAs in Cellular Aging

**DOI:** 10.3389/fendo.2016.00151

**Published:** 2016-12-06

**Authors:** Ufuk Degirmenci, Sun Lei

**Affiliations:** ^1^Institute of Molecular and Cell Biology, Agency for Science, Technology and Research, Singapore; ^2^Department of Biological Sciences, National University of Singapore, Singapore; ^3^Programme in Cardiovascular and Metabolic Disorders, Duke-NUS, Singapore

**Keywords:** senescence, aging, lncRNA, SASP, p21, p53, p16, PRC

## Abstract

Aging is a universal, intrinsic, and time-dependent biological decay that is linked to intricate cellular processes including cellular senescence, telomere shortening, stem cell exhaustion, mitochondrial dysfunction, and deregulated metabolism. Cellular senescence is accepted as one of the core processes of aging at the organism level. Understanding the molecular mechanism underlying senescence could facilitate the development of potential therapeutics for aging and age-related diseases. Recently, the discovery of long non-coding RNAs (lncRNA) provided insights into a novel regulatory layer that can intervene with cellular senescence. Increasing evidence indicates that targeting lncRNAs may impact on senescence pathways. In this review, we will focus on lncRNAs involved in mechanistic pathways governing cellular senescence.

## Introduction

Aging is a time-dependent decline of physiological capabilities. This progressive decay is caused by multiple factors such as the accumulation of senescent cells, mitochondrial dysfunction, disruption of inter/intracellular signaling, and impairment of metabolic homeostasis. Physiological degeneration is the primary risk factor for the age-related diseases such as Alzheimer, cancer, and type-2 diabetes. World population over 60 is expected to double and reach 22% by 2050 while countries like Japan, UK, Germany, and Finland will reach that level in next 5 years, and these statistics are calling for further study of aging biology (1).

Cellular senescence can be activated by mitochondrial dysfunction, chromosome destabilization, telomere attrition, DNA damage, and many other stress factors especially those linked to cell cycle. Clearance of senescent cells (p16^INK4A^ positive cells) was shown to elongate lifespan in mouse models (2), which supports the idea of the cellular senescence as being one of the contributing factors to the organism aging. Other factors such as immune system clearance defect, shorter telomere size, and accumulation of mutations are also pushing forward the physiological decline during aging. However, a consensus view regarding their relative contributions to aging has yet to merge.

Pathologic effects of senescent cells may impact on overall body due to accumulation of them during aging. The emerging evidence suggests that cellular senescence can affect organisms in at least three different stages. First, senescence impairs tissue regeneration due to exhaustion of stem cells. Second, senescent cells release cytokines and chemokines to interact with other cells, which can result in malfunction at tissue or organism level, a phenotype known as senescence-associated secretory phenotype (SASP) (3–9). SASP can explain how relatively low number of cells can cause problems in organs and overall body performance. Third, cellular senescence could intervene with mitochondrial and cellular metabolism through stress activation that results in disturbed energy homeostasis. In contrasts, cellular senescence seems to have a protective function against tumor formation by limiting cellular replication that is consistent with the interception of senescence pathway with tumor response pathways.

Hayflick and Moorehead challenged the cellular immortality idea with their findings on the limit of cellular replication after extensive passaging (10, 11). Later, this senescence type has been linked to telomere attrition, a process that causes chromosomal instability and activates DNA damage response (DDR), which is now known as replicative senescence (4). The DDR pathway is a signal transduction pathway comprised of multiple interacting components. It is initiated by ATM and ATM-Rad3-related (ATR). Downstream of these proteins are checkpoint kinases (CHK1 and CHK2) that phosphorylate and activate downstream effectors such as p53 and p21. Besides telomere attrition, other factors such as UV, oncogenic insult, and chemotherapeutics can also activate DDR and initiate cellular senescence (11–18). In addition, loss of PTEN (tumor suppressor) and overexpression of E2F3 (the S-phase transcription factor) can initiate p53–p21 pathway (19–21).

Senescence activation also occurs through p16^INK4A^, which represses the CDK4–6-mediated phosphorylation of pRB, thereby blocking cell cycle. This pathway may coordinate with the p53–p21 response, depending on stress signal and/or cell types (3, 22–30).

**Figure 1:**
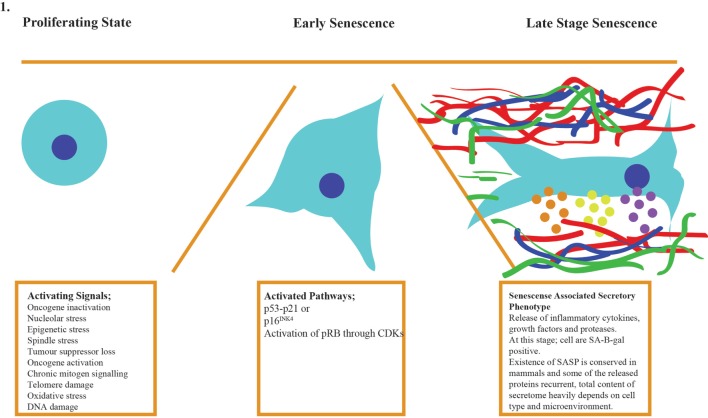
**Cellular senescence occurs in three stages: activation of the signaling cascade, the response of the cell by activation of p53–p21/p16 pathways, and late stage when SAHFs are formed and SASP factors are released to cause organ level malfunctions and pathologies**.

*In vitro* senescent cells have distinctive characteristics such as increased cell size, accumulation of senescence-associated β-galactosidase (SA-β-GAL), activation of survival genes to avoid apoptosis, and the production of the SASP (7, 31–34). The discovery of the SASP provided clues to how senescent cells could disrupt the overall homeostasis of the body albeit their relatively low number. The presence of SASP in mammalian cells appeared to be conserved, and some of the proteins in secretome have been repeatedly detected in multiple cell type. Another senescence-linked phenotype is the formation of senescence-associated heterochromatin foci (SAHFs), which is mainly mediated by p16–pRB pathway. These foci often exist in the genomic loci harboring proliferative genes, thereby inhibiting their expression and activity, but it is important to keep in mind that SHAF does not exist in all senescent cells (35). In senescent cells lacking SAHFs, p16–pRB pathway silences proliferative genes on the epigenetic level as compensatory pathway (36). Currently, measurement of multiple traits mentioned above is the best way to identify senescent cells since each cell type can develop the phenotype with varying order and composition (Figure 1). Figure 1 shows the generic process for cellular senescence while it does not imply the existence of each component in all senescent cells (37–39). Table 1 also indicates specific lncRNAs and their targets.

**Table 1:** **lncRNAs are here listed with their targets, cell type, and organism in which the experiments performed**.

LncRNA name	Target	Cell type	Organism/system
FAL1	p21	Cancer cells	Human/*in vitro*

BANCR	p21	Colorectal cancer cells	Human/*in vitro*

LINCRNA-p21	p21	Mouse embryonic fibroblasts	Mouse/*in vivo* and *in vitro*

MALAT1	p53	Human osteosarcoma cells	Human/*in vitro*

7SL	p53	HeLa cells	Human/*in vitro*

VAD	INK4 locus/p16INK4a	hTERT-immortalized WI38 human fibroblasts	Human/*in vitro*

ANRIL	INK4 locus/p16INK4a	WI38 cell line	Human/*in vitro*

MIR31HG	INK4 locus/p16INK4a	Immortalized human diploid fibroblasts, TIG3-hTERT	Human/*in vitro*

UCA1	p16INK4a mRNA	Mouse primary cells	Mouse/*in vitro*

SAL-RNA	p53	WI38 cell line (old versus young)	Human/*in vitro*

SALNR	NF-90 localization	Human fibroblast cells	Human/*in vitro*

TERRA	Telomere length	Mammalian cells and medulloblastoma	

ASNCMTRNA2	hsa-miR-4485	Human endothelial cells	Human/*in vitro*

HOTAIR	E3 ubiquitin ligase and PRC2	Ovarian cancer cells and HeLa cells	Human/*in vitro*

PANDA	PRC1 and PRC2 recruitment	Primary human diploid fibroblast strains, BJ (neonatal foreskin), and WI38 (fetal lung)	Human/*in vitro*

**Figure 2:**
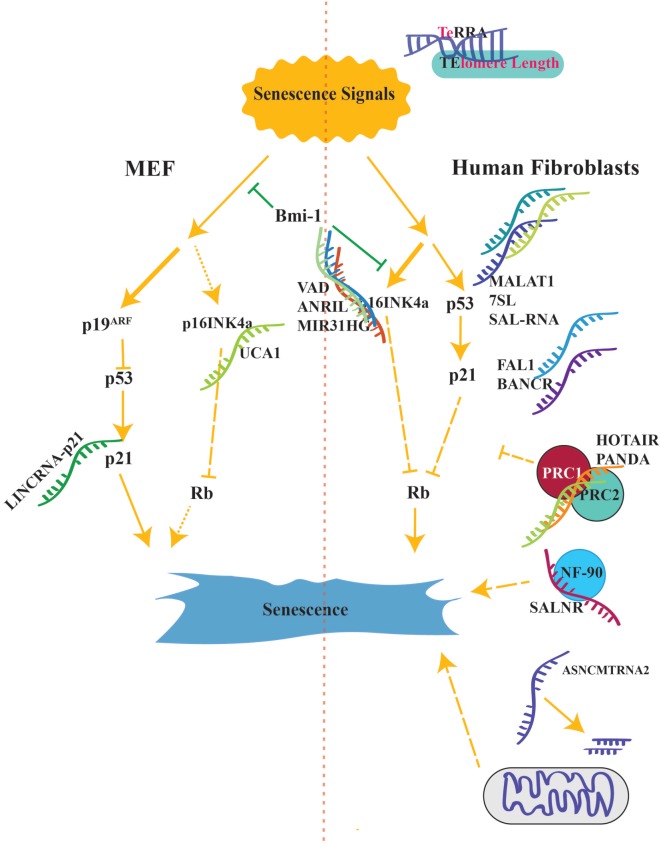
**Senescence activation pathways is shown side by sides from both in human and mouse**. Long lines with small gaps suggest additional unknown intermediates. lncRNAs reviewed in this article have been located as to match their organism. P16^INK4A^ is the major component of senescence in human cells while p19^ARF^ is the main activator in mouse (shown with thicker lines).

*Same list was also used during generation of Figure 2*.

## Emerging Regulatory Layer of Diverse Biological Processes

With protein-coding genes making up to only 2% of the human genome, the other 98% have been considered “junk DNA” till the encyclopedia of DNA elements (ENCODE) project and the functional annotation of the mammalian genome (FANTOM) consortium made their early release. They have challenged this concept by identifying a large number of novel transcripts, namely non-coding RNAs (ncRNAs), from the “junk” regions. Based on their transcript lengths, ncRNAs were further divided into small ncRNAs and long ncRNAs (lncRNAs). lncRNAs are a major class of heterogeneous ncRNAs with the lengths more than 200 nucleotides. They are now being recognized as novel regulators in multiple cellular processes, including development, differentiation, chromosome remodeling, imprinting, and cell cycle control (40–44). Research also shows that lncRNAs are tightly regulated and highly tissue specific (43). Moreover, dysregulation of lncRNAs has been associated with human diseases, such as cancer or neurodegenerative disorders (45). However, the functions of most lncRNAs remain uncharacterized (46, 47).

Long non-coding RNAs often contain a small open reading frame similar to mRNAs, which was a challenge on their distinction (48). Ribosome-profiling experiments have been employed to address this issue. Initial results of ribosome profiling showed that many lncRNAs were, in fact, ribosome-bound while high sensitivity mass spectrometry failed to identify those small peptides from supposed “lncRNA ORFs” (49). With the help of bioinformatics, ribosome release score has been calculated for protein-coding and non-coding RNAs. Ribosome release score is produced by using ribosome dissociation and association values of given part of RNA. Using this score, most lncRNAs fall into the non-coding category, supporting the results of previous mass spectrometry experiments (44, 50).

Regulatory role of lncRNAs in gene expression has been studied. According to these research, their molecular mechanism is quite versatile and there are multiple modes of action (40, 51). lncRNAs may regulate gene expression at transcriptional, RNA processing, translational or posttranslational levels by interacting with DNA, RNA, or protein molecules. The subcellular localization of lncRNAs may also provide additional complexity to their function (52).

Most of the lncRNAs are enriched in the nucleus (43, 53), and their localization supports their suggested role in the epigenetic regulation of the chromatin (54, 55). They function *in cis* to regulate the expression of nearby genes or *in trans* to regulate genes from distant genomic locations. Nuclear lncRNAs often bind to heterogeneous ribonucleoprotein complexes (hnRNPs) and polycomb repressor complex (PRC), consistent with their involvement in modulating epigenetic markers and interacting with transcription factors of target genes. One of the well-known nuclear lncRNAs, Xist, is transcribed from the inactive X chromosome, and it controls dosage compensation of chromosome X in human females (56). Xist recruits polycomb repressive complex 2 (PRC2) (56) that switches histone markers to repressive state, and it inhibits transcription of target genes by blocking the access of RNA polymerase II (RNAPII) to inactivated X chromosome (57, 58). PRCs are one of the major binding partners of lncRNAs that has been identified so far. This affinity is explained by intrinsic promiscuity of PRCs and lncRNAs ability to guide PRCs.

Although lncRNAs are generally enriched in the nucleus, many lncRNAs are also detectable in the cytosol. lncRNAs can control mRNA stability by acting as “sponge” for miRNAs in the cytosol. Sponge lncRNAs compete for the miRNA binding site, which results in rescued expression of mRNAs (59). lncRNAs can also associate with a coding gene by overlapping exons to increase the stability of the target mRNA, e.g., BACE1-AS. In contrast, lncRNAs may also be involved in mRNA degradation through Staufen1 (STAU1)-mediated mRNA decay (SMD). This process recognizes double-stranded RNA for degradation. It has been shown that lncRNAs can form RNA-RNA duplex with 3′-UTRs of target mRNA at Alu elements and cause SMD (60). lncRNA–mRNA complex (bound by Alu element) can be the result of imperfect base pairing, and this double-stranded RNA complex activates SMD, which happens through recruitment of UPF1 (regulator of nonsense transcript-1) that shortens the poly-A tail. Shortened poly-A translates to the shorter lifespan for mRNA (61). Another function is that H19 is transported to the cytosol, where it is used as a source of microRNA (miR-675) to suppress growth (62–71).

In this review, we will focus on the recent studies about lncRNAs involved in cellular senescence (66, 72–77). To incorporate lncRNAs into the known regulatory network, we discussed lncRNAs according to their interactions with known key proteins such as p53/p21, p16, CDKs, and pRb (3–5, 12, 15, 23–25, 27, 28, 30, 78, 79) (Figure 2).

## lncRNAs are Involved in p21 Pathway Regulation (CDKN1A-p21)

Several lncRNAs, as discussed below, have been reported to modulate expression of p21 in different ways. Although the involvement of these p21-regulating lncRNAs in aging has not been directly demonstrated, given the key role of p21 in cellular senescence, it warrants further investigation on how these lncRNAs may participate in cellular senescence regulation through p21.

### Focally Amplified lncRNA on Chromosome 1

Hu et al. focused on somatic copy number alterations (SCNAs) of lncRNAs. By analyzing a genome-wide survey on SCNAs of lncRNA in 2394 tumor specimens from 12 types of cancer, they identified a correlation between copy number of focally amplified lncRNA on chromosome 1 (FAL1) and progression of ovarian cancer. They found out that FAL1 interacts and stabilizes the epigenetic repressor BMI1 (the PRC1 core protein) so as to repress p21 expression. Silencing FAL1 with siRNAs significantly blocked tumor growth *in vivo*. The interaction between FAL1 and BMI1 highlights how lncRNAs can take a central role in epigenetic regulation of p21 (80, 81).

### BRAF-Activated Non-Coding RNA

BRAF-activated non-coding RNA (BANCR) is a lncRNA required for cell migration in different types of cancer (82–84). Shi et al. investigated its contribution to colorectal cancer. They showed that BANCR expression was significantly decreased in colorectal cancer, and when BANCR is overexpressed, it slowed down the growth of colorectal cancer. They also suggested that pcDNA-BANCR-mediated proliferation was linked to activation of G0/G1 cell cycle arrest through regulation of p21. Effects of BANCR were probably post-transcriptional since protein expression levels of p21 increased while that of mRNA remained unchanged. In brief, this article suggests that downregulation of BANCR contributes to the translational regulation of p21 protein (85).

### LincRNA-P21

LincRNA-p21 plays a role in regulating p21 levels. Upon DNA damage, p53 activates lincRNA-p21 that is located next to the p21 gene. In the nucleus, lincRNA-p21 activates transcription of p21 by recruiting hnRNP-K to the promoter region of p21 (86). Using conditional lincRNA-p21 knockout mouse model, Dimitrova et al. showed that loss of lincRNA-p21 could block p21 expression and it can activate mouse embryonic fibroblasts (MEFs) expansion. In the cytoplasm, lincRNA-p21 bound HuR recruits the let-7/Ago2 complex to destabilize lincRNA-p21. LincRNA-p21 also associates with its target mRNAs by base pairing and suppresses their translation with the Rck RNA helicase (86, 87).

## lncRNAs Involved in p53 Regulation

### Metastasis-Associated Lung Adenocarcinoma Transcript 1

The transcript of metastasis-associated lung adenocarcinoma transcript 1 (MALAT1) gene was first identified in 1997 (88), and it is highly expressed in several type of cancers (89). In 2010, MALAT1 was reported as a highly abundant nuclear transcript localized to the nuclear speckles (a region which is enriched in pre-mRNA splicing factors) (90, 91). In 2013, Tripathi et al. performed genome-wide transcriptome analyses in human fibroblasts. Their result indicated that MALAT1 controlled the expression of cell cycle genes and was required for cell cycle progression (92). They also showed that depletion of MALAT1 leads to activation of the p53 pathway and p53 is required for MALAT1 dependent cell cycle arrest. Moreover, MALAT1-depleted cells have low levels of B-MYB (Mybl2, an oncogenic transcription factor), leading to the discovery of aberrant alternative splicing of B-MYB pre-mRNA. However, the exact mechanism of how MALAT1 impacts p53 signaling needs further investigation (91, 92).

### 7SL

In 1991, Sakamoto et al. cloned 7SL sequence partially (93). They observed the 7SL would block proliferation of HeLa cells. Recently, Abdelmohsen et al. showed that 7SL forms a partial hybrid with the 3′-untranslated region (UTR) of p53 mRNA. The association of 7SL with p53 mRNA reduced p53 translation. When 7SL were silenced, it increased the interaction between HuR and TP53 mRNA, stabilizing TP53 mRNA and increasing p53 translation. The antagonist activity between 7SL and HuR for TP53 3′ UTR contributes to the fine tuning of p53 translation (94).

## lncRNAs Regulating CDKN2A

Very long intergenic non-coding RNA-vlincRNA antisense to DDAH1 (VAD) and ANRIL have been identified from senescent cells and found to have an impact on cellular senescence while MIR31HG and UCA1 have been identified through oncogenic pathways.

### Very Long Intergenic Non-Coding RNA-vlincRNA Antisense to DDAH1

Lazorthes et al. have analyzed the differentially expressed strand-specific transcription during oncogene-induced senescence in human. They identified that most vlincRNAs (>50 kb) are induced during senescence. They showed that VAD, one of the antisense vlincRNAs, was highly expressed during senescence and it is essential for maintaining senescence phenotype. VAD regulates chromatin structure *in cis* and activates gene expression *in trans* at the INK4 locus. Moreover, VAD blocks the recruitment of H2A.Z, repressive histone variant, into the proximity of INK4 gene in senescent cells and maintains transcription of it (95).

### Antisense Non-Coding RNA in the INK4 Locus, CDKN2B-AS

Antisense non-coding RNA in the INK4 locus is produced from 9p21 on human chromosome locus where p16^INK4A^ and p15^INK4B^ (p15 in mouse) are transcribed. SNPs in ANRIL gene are linked to various diseases in genome-wide association studies (GWAS). Additionally, silencing of this locus affects cellular proliferation, and it has been found that polycomb complex is recruited to this region by ANRIL *via* SUZ12 (a component of polycomb complex). Silencing of ANRIL produces senescent cells that are stained positive for beta-galactosidase. Current research suggests that ANRIL recruits PRC1 and PRC2 to INK4 region to initiate and maintain silenced chromatin signature: H3K27ME and H2AK119ub1 (96, 97). Therefore, ANRIL maintains the proliferative state by blocking senescence genes.

### MIR31 Host Gene

MIR31 host gene is located 400 kb upstream of the p16^INK4A^ locus on chromosome 9 in human. Previously, decreased expression of MIR31HG has been shown to reduce cell growth and activate strong senescence phenotype (98). Recently, Montes et al. found that the lncRNA MIR31HG was highly expressed in senescence and silencing of MIR31HG activates p16^INK4A^ expression (99). In pre-senescent cells, MIR31HG is located in both nucleus and cytoplasm, but after B-RAF (proto-oncogene) transcription, MIR31HG is specifically present in the cytoplasm. Their results showed that MIR31HG could bind to both p16^INK4A^ and MIR31HG genomic regions. Additionally, polycomb group (PcG) proteins and MIR31HG are necessary for PcG-mediated repression of the p16^INK4A^ locus. Hence, their data suggested a new lncRNA-mediated inhibition of the p16^INK4A^ (99).

### Urothelial Cancer-Associated 1

Kumar et al. discovered that CAPERα/TBX3 repressor complex was required to prevent senescence in primary cells and mouse embryos. CAPERα/TBX3 complex regulates chromatin structure and represses transcription of CDKN2A-p16. Inhibiting CAPERα/TBX3 induced the lncRNA UCA1 expression, in which UCA1 overexpression induced senescence. In proliferating cells, hnRNPA1 can bind and destabilize p16^INK4A^ mRNA. However, during senescence, UCA1 expression stabilizes p16^INK4A^ mRNAs by disrupting the association of hnRNP A1 with p16^INK4A^ mRNA (100, 101).

## Activation of Senescence through Other Pathways

### Senescence-Associated lncRNAs

Abdelmohsen et al. identified differentially expressed novel lncRNAs between young versus old human diploid WI-38 fibroblasts populations. These populations are 32 days apart from each other in doubling time. Among those novel lncRNAs, they selected three lncRNAs to further study their involvement in cellular senescence and proliferation. Decreasing expression of those lncRNAs resulted in increased p53 expression, thereby affecting cell survival and senescence (102). However, exact mechanism requires further study.

### Senescence-Associated Long Non-Coding RNA

Using human fibroblast cells, Wu et al. performed genome-wide screening of lncRNA expression in cellular senescence. They have identified lncRNAs with differential expressions in senescent cells in contrast with young cells. Within these lncRNAs, they further selected a specific senescence-associated lncRNA (SALNR) that has low expression in senescent cells. When they overexpressed the SALNR, cellular senescence was delayed.

Furthermore, they found that SALNR physically interacts with NF90 (nuclear factor of activated T-cells, 90 kDa), which is able to suppress biogenesis of senescence-associated miRNAs, such as miR-22 and miR-181a. Inhibiting NF90 resulted in premature senescence since NF90 is the suppressor of senescence miRNAs.

Moreover, when cells were exposed to Ras-induced stress (activates senescence), NF90 gets translocated to nucleolus and NF90 can no longer suppress senescence-associated miRNA biogenesis, which can be rescued by SALNR overexpression. Thus, SALNR antagonizes NF90 translocation into nucleolus and rescues its inhibitory activity on senescence-associated miRNA expression. Their data suggest that lncRNA SALNR controls cellular senescence through modulating NF90 localization (103).

### Telomeric Repeat-Containing RNA

First, telomeric repeat-containing RNA has been identified in yeast and it has a specific hotspot in the subject of senescence. It forms DNA–RNA pairing with telomere region at R-loop formation that contains G-quadruplex, TERRA, and TERRA-binding proteins. TERRA also increases upon DNA damage at telomere region to involve in recombination and DNA repair. Arnoult et al. showed that TERRA expression decreases upon telomere elongation in multiple cancer cell lines (104). Elongated telomeres have less TERRA expression due to tightly packed telomeric repeats with HP1α and H3K9me3, which make chromosome inaccessible to the transcription machinery.

In parallel, increased levels of TERRA was also found in several primary human tumors containing short telomeres (105). In other words, TERRA is reversely correlated with telomere length.

On a different research, TERRA was shown to maintain the telomere length in the lack of telomerase enzyme as explained above. RNA–DNA complex formed between TERRA and telomeres postpones the senescence by activating homologous recombination at telomeres (106). These suggest multiple functions for TERRA on telomere length and maintenance; however, further research is required to unravel the detailed mechanism of how TERRA functions in telomere and telomerase during aging (107–109).

## Mitochondrial lncRNA Involved in Senescence

### ASNCMTRNA2 (mtDNA-Transcribed Long-Non-Coding-RNA-2)

Villegas et al. have first reported a mitochondrial transcript of 2374 nucleotides whose expression correlates with the cellular replication (110). Following up this project, Burzio et al. have reported the existence of two antisense mitochondrial transcripts that were expressed in proliferating cells from healthy human tissues but decreased in tumor samples (111). Recently, Bianchessi et al. showed that two miRNAs, hsa-miR-4485 and hsa-miR-1973, have perfect homology with ASncmtRNA-2. Both miRNAs are upregulated in endothelial cells at replicative senescence. ASncmtRNA-2 overexpression results in an increased expression of hsa-miR-4485. It induces cell cycle arrest at G2/M phase. Moreover, the overexpression of the two miRNAs shows the similar phenotype, cell cycle delay in both the G1 and G2/M phases. They proposed that ASncmtRNA-2 is involved in replicative senescence in endothelial cells by causing cell cycle arrest in the transition from G2/M to G1 (112).

## Maintenance of Senescence

### HOX Transcript Antisense RNA

The human lncRNA HOX transcript antisense RNA (HOTAIR) is transcribed from the HOXC locus of homeobox genes as an approximately 2.2-kb RNA (113). It binds to the enhancer of zeste homolog 2 (EZH2) in the polycomb repressive complex 2 (PRC2) and to lysine-specific demethylase 1 (LSD1), which are chromatin remodeling factors (113, 114). Both complexes lead to gene silencing and are recruited in *trans* to hundreds of genomic sites by HOTAIR. Recently, Ozes et al demonstrated a novel function of HOTAIR in DDR, which is regulating the expression of genes to block proliferation. Their results showed that after induction of DNA damage, HOTAIR activates the cellular senescence through NF-κB pathway. Furthermore, they demonstrated the presence of a positive-feedback loop that induces HOTAIR. This activates DDR and sustains NF-κB expression (115). Recently, Yoon et al. reported a function of lncRNA HOTAIR as a scaffold for ubiquitin-mediated proteolysis in posttranslational control (116). First, HOTAIR binds to E3 ubiquitin ligases through RNA-binding domains, Dzip3 and Mex3b, and to their ubiquitination targets: Ataxin-1 and Snurportin-1, respectively. As a result, HOTAIR assists the ubiquitination of Ataxin-1 by Dzip3 and Snurportin-1 by Mex3b and speeds up their degradation. HOTAIR expression is increased in senescent cells, resulting in the degradation of Ataxin-1 and Snurportin-1 (116). Their results suggested a novel scaffold function for a lncRNA that can impact on senescence.

### P21-Associated ncRNA DNA Damage Activated

With the help of super high-resolution tiling array analysis in CDKN1 region, Puvvula et al. identified P21-associated ncRNA DNA damage activated (PANDA) as a lncRNA, which is induced upon DNA damage.

P21-associated ncRNA DNA damage activated plays a dual role in proliferating cells versus senescent cells. In proliferating cells, scaffold-attachment-factor A/hnRNPU (SAFA) interacts with lncRNA PANDA to block senescence genes by recruiting PRC1 and PRC2 to senescence activating genes loci including CDKN1. Silencing SAFA and PANDA induces cell cycle arrest, and it also causes senescence in proliferating cells by allowing transcription of senescence activating genes.

In senescent cells, SAFA–PANDA–PRC interactions are blocked therefore, pro-senescence genes are transcribed. PANDA inhibits the expression of proliferative genes by sequestering the transcription factor NF-YA from occupying its target promoters in senescent cells. Depletion of PANDA in the senescent cells reactivates proliferative genes and allows cells to replicate (117).

## Conclusion and Perspectives

As concluding remarks, with the recent advancement in technology, our understanding on the function of lncRNAs has expanded vastly. Here, we have summarized an emerging role of lncRNAs as regulators of cellular senescence and age-related diseases. Albeit of these progresses, lncRNAs in senescence field is still in its infancy so as lncRNAs in general. Most of these studies remain at the cellular level, but the evidence that lncRNAs can regulate aging process *in vivo* has yet to be established.

Understanding lncRNAs function may help us fill some knowledge gap in genetic causes for human phenotypes. Many SNPs are not associated with coding genes but with non-coding region where lncRNAs may be transcribed (118). For example, a recent study on Asian centurions revealed a novel SNP rs2440012 associated with longevity (119). Given the growing evidence of the functional role lncRNAs in physiological and pathological processes, we believe it is just a matter of time to illustrate role of lncRNAs in aging.

## Author Contributions

UD wrote the manuscript. SL reviewed and edited the manuscript.

## Conflict of Interest Statement

The authors declare that the research was conducted in the absence of any commercial or financial relationships that could be construed as a potential conflict of interest.

## Abbreviations

CDKN1, cyclin-dependent kinase inhibitor 1; CHK, checkpoint kinase; DDR, DNA damage response; GWAS, genome-wide association study; hnRNP, heterogeneous nuclear ribonucleoproteins; lncRNA, long non-coding RNA; MEF, mouse embryonic fibroblast; ORF, open reading frame; PRC, polycomb repressor complex; SAHF, senescence-associated heterochromatin foci; SASP, senescence-associated secretory phenotype; SCNA, somatic copy number alterations; SMD, staufen1 (STAU1)-mediated mRNA decay; UTR, untranslated region.
